# Endogenous glucagon-like peptide- 1 and 2 are essential for regeneration after acute intestinal injury in mice

**DOI:** 10.1371/journal.pone.0198046

**Published:** 2018-06-04

**Authors:** Rasmus Hytting-Andreasen, Emilie Balk-Møller, Bolette Hartmann, Jens Pedersen, Johanne Agerlin Windeløv, Jens Juul Holst, Hannelouise Kissow

**Affiliations:** 1 Department of Biomedical Sciences, University of Copenhagen, Copenhagen, Denmark; 2 NNF Center of Basic Metabolic Research, University of Copenhagen, Copenhagen, Denmark; National Institutes of Health, UNITED STATES

## Abstract

**Objective:**

Mucositis is a side effect of chemotherapy seen in the digestive tract, with symptoms including pain, diarrhoea, inflammation and ulcerations. Our aim was to investigate whether endogenous glucagon-like peptide -1 and -2 (GLP-1 and GLP-2) are implicated in intestinal healing after chemotherapy-induced mucositis.

**Design:**

We used a transgenic mouse model Tg(GCG.DTR)(Tg) expressing the human diphtheria toxin receptor in the proglucagon-producing cells. Injections with diphtheria toxin ablated the GLP-1 and GLP-2 producing L-cells in Tg mice with no effect in wild-type (WT) mice. Mice were injected with 5-fluorouracil or saline and received vehicle, exendin-4, teduglutide (gly2-GLP-2), or exendin-4/teduglutide in combination. The endpoints were body weight change, small intestinal weight, morphology, histological scoring of mucositis and myeloperoxidase levels.

**Results:**

Ablation of L-cells led to impaired GLP-2 secretion; increased loss of body weight; lower small intestinal weight; lower crypt depth, villus height and mucosal area; and increased the mucositis severity score in mice given 5-fluorouracil. WT mice showed compensatory hyperproliferation as a sign of regeneration in the recovery phase. Co-treatment with exendin-4 and teduglutide rescued the body weight of the Tg mice and led to a hyperproliferation in the small intestine, whereas single treatment was less effective.

**Conclusion:**

The ablation of L-cells leads to severe mucositis and insufficient intestinal healing, shown by severe body weight loss and lack of compensatory hyperproliferation in the recovery phase. Co-treatment with exendin-4 and teduglutide could prevent this. Because both peptides were needed, we can conclude that both GLP-1 and GLP-2 are essential for intestinal healing in mice.

## Introduction

Mucositis in the oral cavity and the gastrointestinal (GI) tract is a common side effect in patients receiving chemotherapy. Elting et al. 2003 [1] reported that in approximately 50% of cancer patients oral and/or GI mucositis developed during a chemotherapy treatment period, and 11% of patients needed total parenteral nutrition (TPN) [1]. The symptoms of GI mucositis include diarrhoea, dehydration, abdominal pain and increased systemic infection rate due to inflammation and ulceration of the mucosa[1]. Severe GI mucositis can have clinical and economical implications, including need for TPN, hospitalisation, opioid usage and reduction in chemotherapy drug dose [1–3]. There is currently no method to prevent severe GI mucositis and the current treatment consists of symptom relief, antibiotics and rehydration. The underlying mechanism for the development of mucositis is not fully understood, but Sonis et al. 2004 suggested a model with five phases, ending with healing of the GI tract as a consequence of continued proliferation and regeneration of the intestinal epithelium [3;4].

The intestinal hormones glucagon-like peptide- 1 (GLP-1) and glucagon-like peptide-2 (GLP-2) are co-secreted from the enteroendocrine L-cells in response to food intake [5;6]. GLP-2 is important for the intestinal crypt cell proliferation and regulation of mucosal apoptosis and improves the barrier function of the small intestine (SI) [7–10]. It has been claimed to work indirectly through ErbB, keratinocyte growth factor and insulin-like growth factor 1 [11–14], and in support of these findings the GLP-2 receptor (GLP-2R) was found to be expressed in subepithelial myofibroblast, as well as in endocrine cells [9;12;13]. GLP-1, the well-known anti-diabetic sister hormone to GLP-2, was in 2007 shown to have a trophic effect in the rat SI [15] as well as in mice [16] and was shown later on to ameliorate GI mucositis also in mice [17]. Furthermore, it was shown that plasma levels of both GLP-1 and GLP-2 were elevated in rats and mice with mucositis [17]. These findings lead us to the hypothesis that the endogenous hormones are essential for intestinal healing. The consequence of this could be insufficient healing and regeneration in patients with impaired endogenous secretion either because of a lack of stimulation (fasting) or lack of secretion, which is documented in obese patients (body mass index ≥ 30)[18], and those with type 2 diabetes [18–21]. We aimed to investigate the role of endogenous GLP-1 and GLP-2 for the recovery of the SI after chemotherapy-induced injury. We studied the natural history of 5-fluorouracil (5-FU)-induced mucositis in mice and showed that co-treatment with the two hormones outperformed single treatment of mucositis. We used a GLP-1 and GLP-2 cellular knockout mouse model (Tg(GCG.DTR))[22] to demonstrate that the endogenous secretion of both hormones is essential for intestinal recovery and that substitution with *both* hormones is necessary to regain normal recovery after chemotherapy-induced mucositis.

## Materials and methods

### Animals

The animal studies were approved by the Danish National Committee for Animal Studies (permit number 2013-15-293400833). Female CD1 mice (Charles River, Germany) (25–30 g) were used in study 1 and female C57BL/6J mice (Charles River, Germany) (20–25 g) were used in studies 2 and 3. Female Tg(GCG.DTR)(Tg) mice (20–25 g) described by Pedersen et al.[22] were used in studies 4 and 5. The Tg mouse has the human diphtheria toxin receptor inserted into the *Gcg* gene. This strain develops deficiency for proglucagon-derived peptides, when diphtheria toxin (DT) (1 mg/kg dissolved in 0.1% bovine serum albumin (BSA)) is administered intraperitoneally (i.p.) every second day. Wild-type littermates injected with DT served as a control (WT). To ensure that the DT did not cause a change in phenotype between Tg and WT, experiments replacing DT with BSA were also performed (S1 Appendix). All animals were housed at the animal facility at the Panum Institute, Copenhagen, Denmark. They were kept in temperature- (21°C) and humidity- (55%) controlled rooms with light/dark cycles of 12 h in individually ventilated cages. Throughout the study, they were maintained on water and chow (no. 1314, Altromin, Germany) ad libitum. Body weight (BW) was recorded daily. Mice did not receive analgesia during the experiments, but to alleviate suffering mice with severe BW loss were sacrificed. In all circumstances sacrifice implied terminal bleeding in full anaesthesia, followed by opening of the thoracic cavity.

### Induction of mucositis

Mucositis was induced with a single i.p. injection (400 mg/kg) of 5-fluorouracil (5-FU; Hospira Nordic AB, Sweden). Saline was used in animals serving as healthy controls.

*Peptides*: The mice were treated twice daily by subcutaneous injections with either the GLP-1 analogue exendin-4 (Ex-4) (12.5 μg/animal) and/or the GLP-2 analogue teduglutide (aGLP-2) (12.5 μg/animal) (CASLO Aps, Lyngby, Denmark). In experiments where mucositis was induced, the treatment was initiated 2 days prior to chemotherapy. Peptides were dissolved in phosphate-buffered saline (PBS) containing 10% Haemaccel (Behringwerke AG, Marburg, Germany). PBS with 10% Haemaccel served as control (vehicle).

### Sample collection

On the day of sacrifice, the mice were anaesthetised with ketamine (Intervet International B.V. (Holland)/xylazine (Bayer Animal, Germany) 100/10 mg/kg i.p. After anaesthesia, the mice were weighed and the abdomens were opened; blood was drawn from the inferior vena cava and collected in 1.5 mL EDTA Eppendorf tubes (2 mg/mL, Milian Dutscher Group, Switzerland) containing a DPP-4 inhibitor (valine-pyrrolidide 0.01 mmol/L, Novo Nordisk, Denmark), and tubes were then kept cold. The small intestines were removed, flushed with saline, excess saline carefully removed and the outer surface dried with paper before the weight was recorded. Samples from the duodenum, the jejunum and the ileum were collected and fixed in 4% paraformaldehyde in PBS pH 7.2 (VWR, Radnor, Pennsylvania, USA) for histology. Samples from the duodenum and the ileum were collected and snap frozen for myeloperoxidase (MPO) activity (studies 3–5).

### GLP-2 concentration in plasma

The blood samples were centrifuged at 1200 g for 15 min at 4°C and plasma stored at -20°C. The plasma was extracted with 75% ethanol before analysis and active GLP-2 was measured by radioimmunoassay as described by Hartmann et al. 2000 [23] using an N-terminal specific antiserum (code 92160, in-house) (studies 1 and 4).

### Histology

Fixed tissue was dehydrated in 70% ethanol and embedded in paraffin. The tissues were cut in 5- μm transverse sections and stained with hematoxylin and eosin and examined with a light microscope connected to a camera (Zeiss AxiosTgp 2 plus, Brock & Michelsen, Birkeroed, Denmark). The morphological parameters (mucosal area, villus height, crypt depth) were analysed using the software Zen 2 (blue edition) (Carl Zeiss Microscopy GmbH, Göttingen, Germany).

Grades of mucositis were evaluated using a modified version of a method described by Howarth et al. [24] (studies 3–5). Scores between 0 and 5 were given for the four intestinal compartments; submucosa and muscularis (thickening and/or oedema), lamina propria (infiltration of inflammatory cells and/or dilatation of lymphatic vessels), crypts (loss of crypts, architectural disruption, crypt abscess) and villi (fusion, stunting, disruption of enterocytes). Scores from each compartment were added to a total mucositis score and results are given as a mean for all scores in the three segments of the intestine. All measurements and evaluations were performed blinded from the observer.

Proliferation analysis (study 1) was performed by injecting the animals with BrdU (5′-bromo-2′-deoxyuridine) 50 mg/kg i.p. 150 min before sacrifice. Immunohistochemistry was performed in sections from the duodenum, ileum and jejunum. For antigen retrieval, sections were incubated in citrate buffer (pH 6.0) in a microwave oven at 750 W for 15 min. The sections were preincubated in 2% BSA for 10 min, followed by an incubation with a 1:200 diluted monoclonal mouse anti-BrdU antibody Ab-4(Bu20a) (Thermo Scientific, USA)(overnight). The sections were incubated for 40 min with a 1:200 dilution of biotinylated horse anti-mouse IgG (Vector BA-2000 Vectos Laboratories, USA), followed by StreptAB complex/horseradish peroxidase (Vectastain ABC kit, Vector Laboratories, USA). The sections were developed with 3,3’-diaminobenzidine containing nickel ammonium sulphate (Sigma-Aldrich, Denmark) for 15 min. The area of the BrdU immunoreactive cells per crypt was measured in 10 crypts per section using the software Image Pro 7.0 (Media, Cybernetics, USA).

### Myeloperoxidase

A modified version of the study by Binker et al. [25] was used to assess the activity of MPO as a marker of neutrophil infiltration (studies 3–5). The tissue was weighed and mixed with 0.5 mL 50 mM K- phosphate buffer pH 6.0 with 50 mM hexadecyltrimethylammonium bromide (Sigma-Aldrich, Denmark) in 2 mL Eppendorf tubes containing a 5-mm stainless steel bead (Qiagen, Germany). Samples were homogenised for 6 min at 25 Hz (Qiagen TissueLyser System), and snap frozen on dry ice followed by thawing in water. This step was repeated three times. Samples were centrifuged (16,000 g, 30 min, at room temperature). Supernatants were diluted in 50 mM K-phosphate buffer (pH 6.0) and 10-μL samples were loaded onto a 98-well plate with 190 μL substrate buffer (50 mM K-phosphate buffer, 0.167 mg/mL O-dianisidine dihydrochloride and 0.0005% hydrogen peroxide (H_2_O_2_)). The change in absorbance was measured at 450 nm every minute. Changes were checked for linearity and units of MPO/mg were calculated using the extinction coefficient for H_2_O_2_ (1.13 x 10^4^ M^-1^ cm^-1^) defining one unit as the amount of enzyme that degrades 1 μmol H_2_O_2_/min.

### Study setups

#### Study 1 –The natural history of mucositis induced with 5-FU

To establish the time point of the acute phase and the recovery phase of mucositis, mice were injected with 5-FU and divided into six groups representing day 0 to 5 (n = 5) and one of the groups sacrificed each day for 5 consecutive days. The mice sacrificed at day 0 had an injection with saline and served as healthy controls. Endpoints were BW (recorded at day 0 and the day of sacrifice in each mouse), SI weight, morphology, proliferation and plasma concentration of GLP-2.

#### Study 2 –Single vs. co-treatment with Ex-4 and aGLP-2 in normal healthy mice

Mice were divided into four groups: Group 1: Ex-4; group 2: aGLP-2; group 3: co-treatment (Ex-4 and aGLP-2); group 4: saline (n = 8). Mice were treated for 10 days. Endpoints were BW, SI weight and morphology.

#### Study 3—Single vs. co-treatment with Ex-4 and aGLP-2 of acute mucositis

Mice were divided into five groups: Group 1: 5-FU and Ex-4; group 2: 5-FU and aGLP-2; group 3: 5-FU co-treatment (Ex-4 and aGLP-2); group 4: 5-FU vehicle; group 5: saline vehicle (n = 8). 5-FU was injected on day 0 and the mice were sacrificed on day 3 (acute phase). Endpoints were BW, SI weight, morphology, grade of mucositis and MPO.

#### Study 4—Recovery of mucositis in GLP-1 and GLP-2 deficient mice

Tg and WT mice were divided into four groups: group 1: Tg 5-FU; group 2: WT 5-FU; group 3: Tg saline; group 4: WT saline (n = 8). 5-FU was injected on day 0 and the mice were sacrificed on day 5 (recovery phase). Endpoints were BW, SI weight, morphology, grade of mucositis and MPO. Plasma GLP-2 was measured to ensure insufficient L-cell secretion upon mucositis.

#### Study 5 –Single vs. co-treatment with Ex-4 and aGLP-2 in GLP-1 and GLP-2 deficient mice with mucositis

Tg or WT mice were divided into six groups: group 1: Tg 5-FU vehicle; group 2; Tg 5-FU Ex-4; group 3: Tg 5-FU aGLP-2; group 4: Tg 5-FU co-treatment (Ex-4 and aGLP-2); group 5: WT 5-FU vehicle; group 6: WT saline vehicle (n = 3–9). 5-FU was injected on day 0. The mice were sacrificed on day 5 (recovery phase). Endpoints were BW, SI weight, morphology, grade of mucositis and MPO.

### Statistics

All results are shown as means and standard error of the mean (SEM). Comparison between groups in SI weight, GLP-2 plasma levels, proliferation, villi length, crypt depth, mucositis score, MPO activity and BW in study 1 were performed with an analysis of variance (ANOVA) followed by a Dunnett or Bonferroni multiple comparisons test. BW progression data (studies 2–5) were analysed by a two-way ANOVA followed by a Bonferroni multiple comparisons test. All analyses were performed using GraphPad Prism V5.01, and probability values p<0.05 were considered significant.

## Results

### The nature of mucositis induced with 5-FU

**Fig 1:**
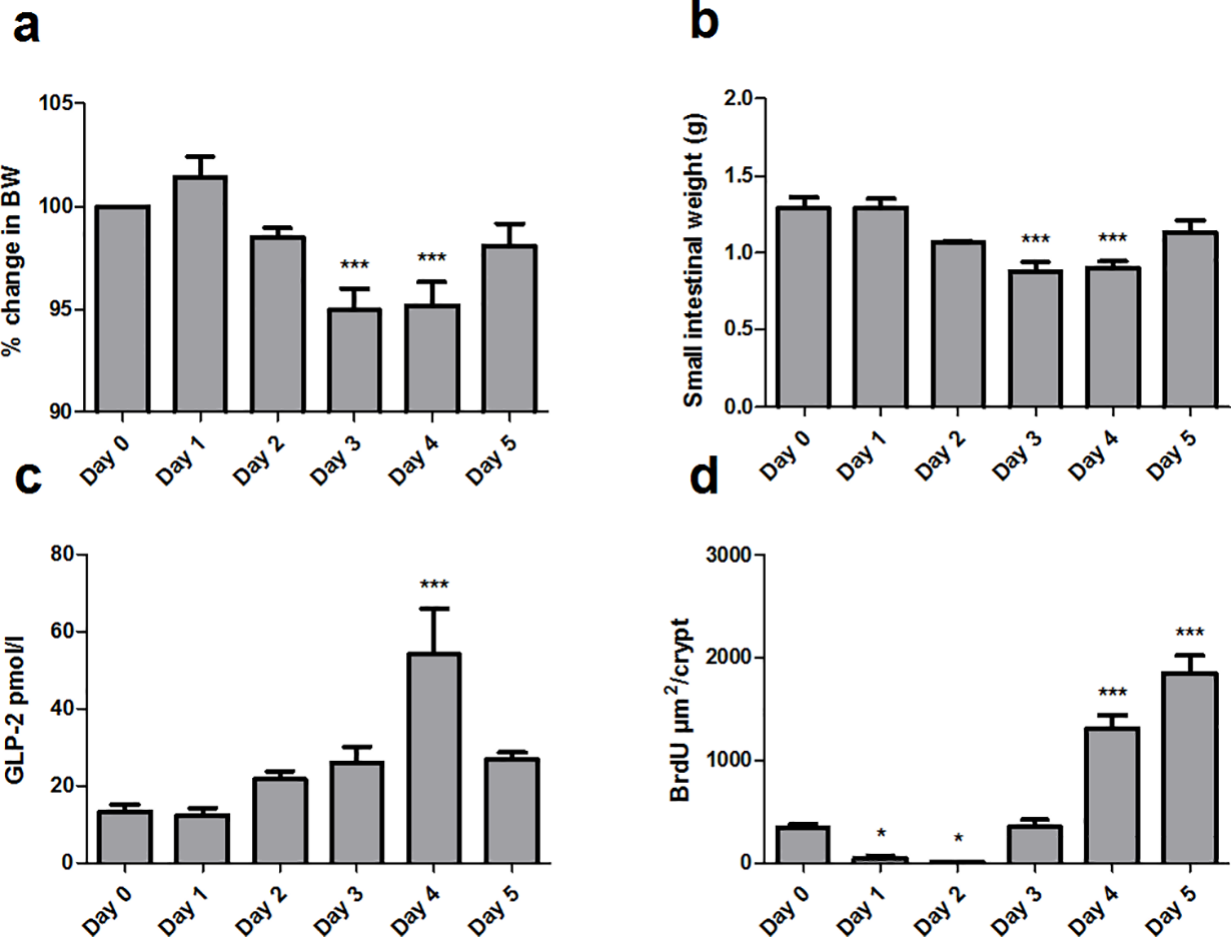
The nature of mucositis induced with 5-FU. Mice were sacrificed 1 to 5 days after 5-FU injection at day 0. **a** Percent change in BW from day 0, **b** small intestinal weight (g), **c** area of BrdU immunopositive cells/crypt (μm2) in ileum, **c** plasma concentration of GLP-2 (pmol/L). Results are shown as mean ± standard error of the mean. n = 13. *p<0.05, ***p<0.001 compared to day 0 (ANOVA followed by Dunnett multiple comparison test).

At 3 and 4 days after 5-FU injection, the mice suffered a significant loss in BW and SI weight (p<0.001), but mice sacrificed 5 days after 5-FU had almost regained their starting BW and SI weight (Fig 1). We measured the area of BrdU positive cells/crypt as an estimate for proliferation (Fig 1D). We found a significant decrease on day 1 and day 2 (as expected), at day 3 the proliferation was comparable to day 0 but by day 4 and day 5 the proliferation increased remarkably. This corresponded to an increase in plasma concentration of GLP-2, which was elevated approximately threefold at day 4 (54 pmol/L ± 17vs. 13 pmol/L ± 7) (Fig 1D and S1 Fig). Morphology confirmed that the hyperproliferative response at days 4 and 5 could be seen as increased crypt depth (S1 Fig).

### Co-treatment with Ex-4 and aGLP-2 exerts synergistic trophic effects in normal healthy mice

**Fig 2:**
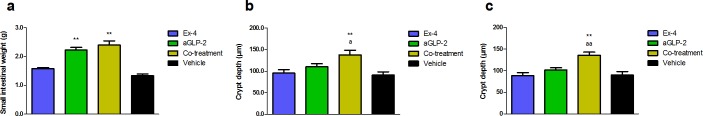
Co-treatment with Ex-4 and aGLP-2 exerts synergistic trophic effects in normal healthy mice. **a** Small intestinal weight (g), **b** crypt depth in duodenum, **c** crypt depth in jejunum.Results are shown as mean ± standard error of the mean. n = 8. **p<0.01 compared to vehicle, a = p<0.05, aa = p<0.01 compared to aGLP-2 treatment (ANOVA followed by Dunnett or Bonferroni multiple comparison test).

On the first day after the beginning of treatment, mice treated with Ex-4 (Ex-4 group and co-treated group) had a small but significant weight loss (presumably due to the anorectic effect of GLP-1 agonists); however, the mice quickly regained their BW (S2 Fig). The SI weight was significantly (p<0.01) increased in the aGLP-2 and co-treated mice compared to the vehicle-treated mice (Fig 2). Single treatment with Ex-4 or aGLP-2 in the chosen doses did not lead to an increased crypt depth in duodenum and jejunum, but when the peptides were administered simultaneously, there was a significant trophic effect (Fig 2).

### Co-treatment with Ex-4 and GLP-2 outperforms single treatment in a model of acute mucositis

**Fig 3:**
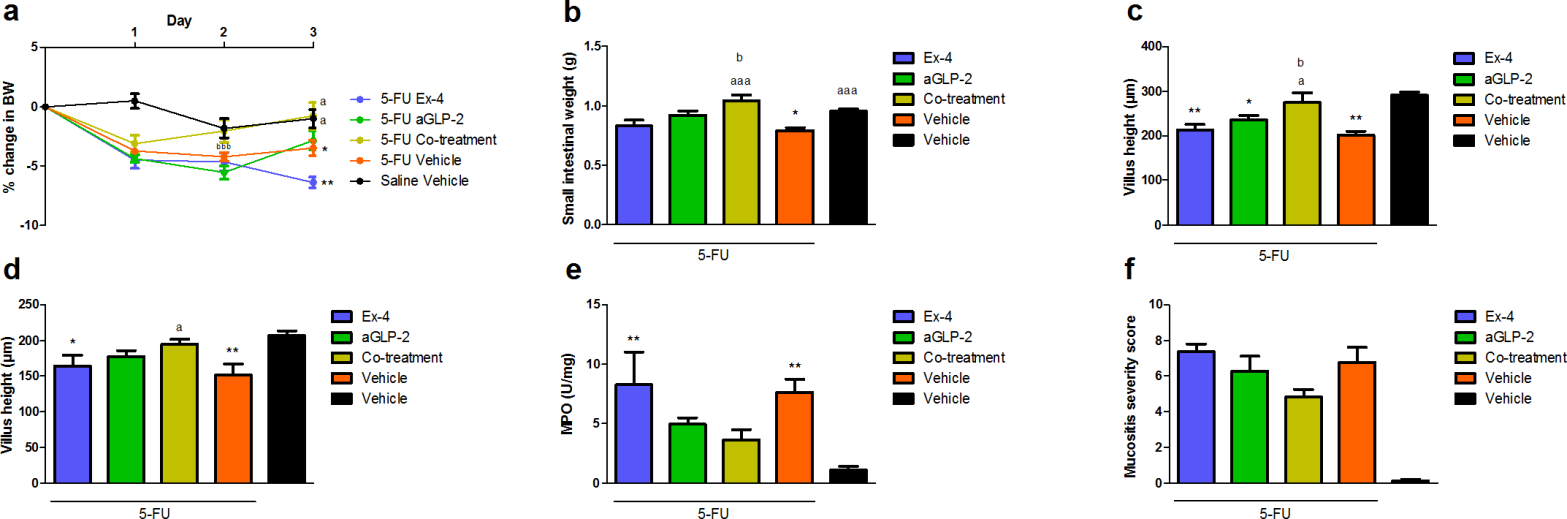
Co-treatment with Ex-4 and GLP-2 outperforms single treatment in a model of acute mucositis. **a** Percent change in BW from induction of mucositis, **b** small intestinal weight (g), **c** villus height in jejunum (μm), **d** villus height in ileum (μm), **e** activity of MPO in ileum (U/mg), **f** mucositis severity score in small intestine. Results are shown as mean ± standard error of the mean. n = 6–8. *p<0.05, **p<0.01, ***p<0.001 compared to healthy control (vehicle) a = p<0.05, aa = p<0.01, aaa = p<0.001 compared to 5-FU vehicle and b = p<0.05, bb = p<0.01 compared to aGLP-2 (two-way ANOVA followed by Bonferroni multiple comparison test (BW) or ANOVA followed by Dunnett or Bonferroni multiple comparison test).

Within the first 24 h after chemotherapy injection all animals lost approximately 5% of their BW, but only vehicle and Ex-4 treated mice had a significant final BW loss compared to the healthy controls (Fig 3). Vehicle-treated mice had a significant decrease in SI weight, a decrease in jejunal and ileal villus height, and an increase in ileal MPO activity 3 days after chemotherapy: only co-treatment could significantly prevent this (Fig 3). When comparing aGLP-2 treatment with co-treatment we found that co-treatment prevented the BW loss from day 2 (this was highly significant during aGLP-2 treatment alone) and that SI weight and villus height in jejunum were significantly larger in co-treated compared to aGLP-2 treated animals. Co-treatment also tended to prevent the increase in mucositis severity score; however, this did not reach statistical significance (Fig 3). Detailed morphology data are shown in supplementary data (S3 Fig).

### GLP-1 and GLP-2 deficient mice fail to regenerate after acute mucositis

**Fig 4:**
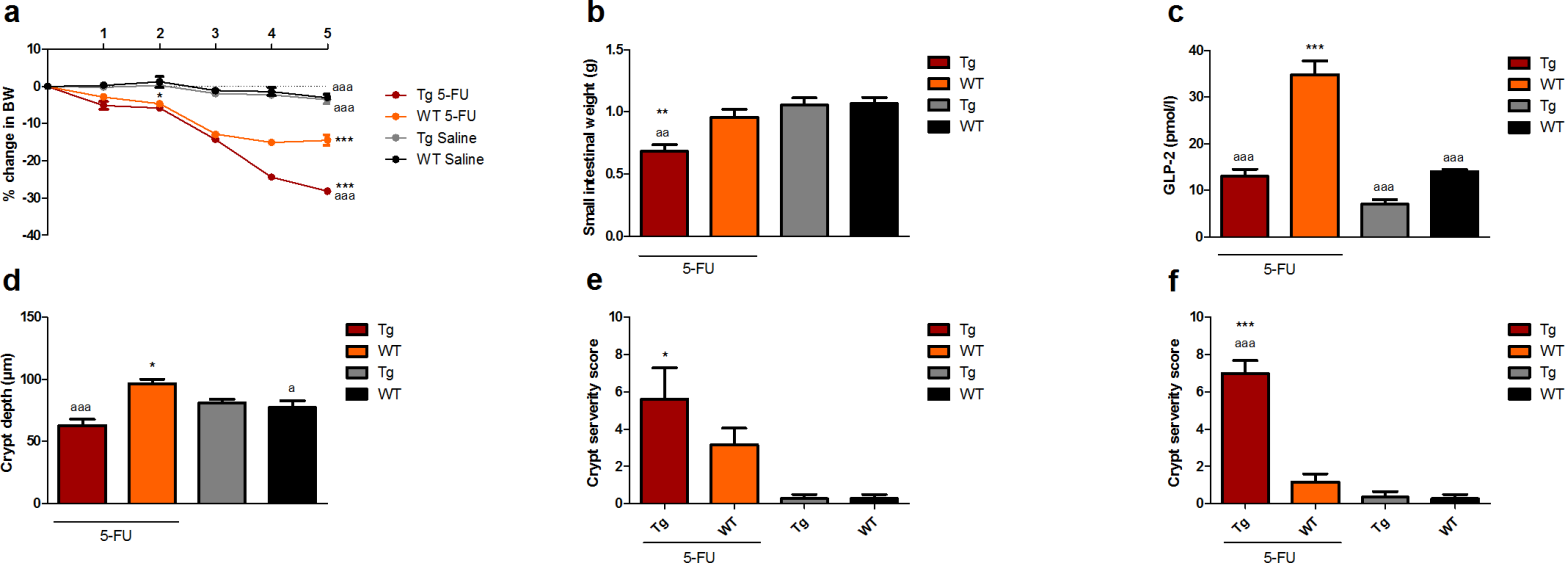
GLP-1 and GLP-2 deficient mice fail to regenerate after acute mucositis. **a** Per cent change in BW, **b** small intestinal weight (g), **c** plasma concentration of GLP-2 (μmol/L) **d** crypt depth in ileum (μm), **e** crypt severity score in jejunum, **f** crypt severity score in ileum. Results are shown as mean ± standard error of the mean. n = 4–8. *p < 0.05, **p < 0.01, ***p<0.001 compared to healthy control (WT saline), a = p < 0.05, aa = p < 0.01 aaa = p<0.001 compared to WT 5-FU (two-way ANOVA followed by Bonferroni multiple comparison test (BW) or ANOVA followed by Dunnett multiple comparison test).

GLP-1 and GLP-2 deficient mice had a severe loss of BW and decrease in SI weight in the recovery phase, highly significant from the WT 5-FU mice (p<0.01) (Fig 4). Normally the GLP-2 levels are elevated after chemotherapy injection [17;26;27], and to ensure that the Tg mice had insufficient GLP-2 secretion we measured the GLP-2 levels. In contrast to the WT mice the Tg mice did not secrete GLP-2 as a consequence of mucositis (Fig 4). Furthermore, Tg mice failed to show compensatory hyperproliferation, and crypts were significantly shorter than in both WT mice and healthy controls (Fig 4). Detailed morphology data are shown in supplementary data (S4 Fig). We did not find any significance in the levels of MPO or total severity score between the Tg and WT groups (results not shown), but when examining the crypt compartment the Tg mice still suffered from the acute damage with loss of crypts, architectural disruption and crypt abscesses. This finding was highly significantly different from the WT mice, which were comparable to control animals without induction of mucositis.

### Co-treatment with Ex-4 and aGLP-2 are pivotal for regeneration after acute mucositis in L-cell deficient mice

**Fig 5:**
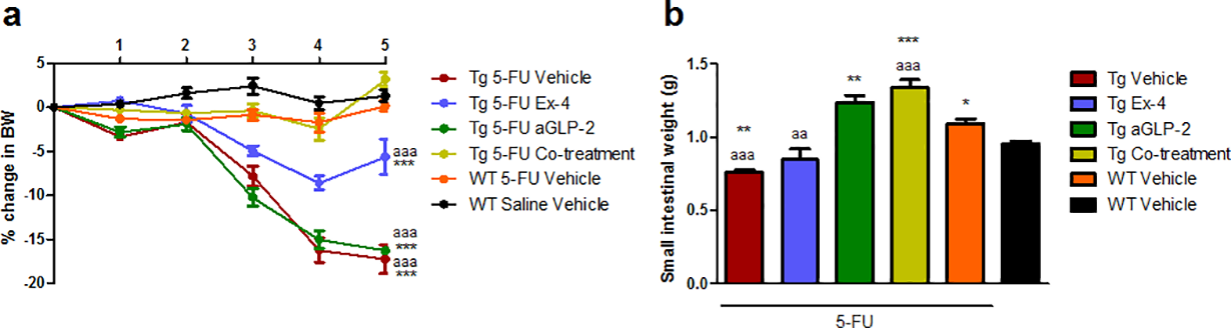
Co-treatment with Ex-4 and aGLP-2 are pivotal for regeneration after acute mucositis in L-cell deficient mice. **a** Percent change in BW and **b** small intestinal weight (g) 5 days after induction of mucositis. Results are shown as mean ± standard error of the mean. n = 3–12. *p < 0.05, **p < 0.01, ***p<0.001 compared to healthy control (WT saline vehicle). aa = p < 0.01, aaa = p<0.001 compared to Tg 5-FU vehicle (two-way ANOVA followed by Bonferroni multiple comparison test (BW) or ANOVA followed by Dunnett multiple comparison test).

Only co-treatment and treatment with exendin-4 could significantly prevent the severe BW loss in Tg mice after chemotherapy (Fig 5). As in study 4, Tg mice failed to recover from the SI weight loss, but aGLP-2 and co-treatment significantly prevented this (Fig 5). The morphology confirmed that treatment with aGLP-2 alone or co-treatment was able to rescue the loss in mucosa mass by showing clear signs of hyperproliferation (S5 Fig). Histological severity scores were generally low because the intestines were scored 5 days after injury, but with Tg mice scoring highest in all segments (S5 Fig). Again, no differences were seen regarding the measurements of MPO (results not shown).

## Discussion

GLP-1 and GLP-2 are products of the pro-glucagon gene [28]. The pro-glucagon gene is expressed primarily in the α-cells in the endocrine pancreas [29] and in the enteroendocrine L-cells in the intestine [30]. Differences in the posttranslational processing of pro-glucagon give rise to tissue-specific products of the gene [31]. In the pancreatic α-cells, posttranslational processing gives rise to glucagon, whereas the L-cell secretes GLP-1 and GLP-2 in an equipotent manner. GLP-2 is a well-known growth factor for the intestinal epithelia; the hormone is considered to be responsible for intestinal adaptation by stimulating proliferation and inhibiting apoptosis [32;33]. The main action of GLP-1 is to stimulate insulin in a glucose-dependent manner [34]; but recently it has been shown that injections with GLP-1 analogues cause both small intestinal and colonic growth in rats [15] and mice [16;35]. The stimulatory signal for secretion has been intensively investigated, and it is clearly meal-related [36]. A previous study showed that fasting for 48 h in rats led to SI atrophy but this was reversed following a period of 48 h of re-feeding. Furthermore, this re-feeding adaptation was abolished upon antagonism of GLP-2r [37]. Similar responses were reported in mice [9]. Likewise, it is well known that rodents and pigs receiving TPN experience severe intestinal atrophy [37;38], which can be ameliorated by co-infusion with GLP-2 [38;39]. However, patients with active inflammatory bowel disease were found to have higher levels of GLP-2 [40] and GLP-1 [41], and together with our earlier findings that both hormones were markedly increased in rats 2 days after 5-FU [17;27], it seems that the hormones are also secreted in response to intestinal injury.

In the current study, we first decided to establish the natural course of mucositis induced with 5-FU in mice. 5-FU is transformed into cytotoxic metabolites, which are incorporated into DNA leading to apoptosis by inhibiting DNA synthesis [42]. 5-FU prevents the renewal of the surface epithelium of the villi, which will lead to lowering of the epithelial integrity and influx of inflammatory cytokines. The chemotherapy-induced intestinal injury is followed by recovery of the mucosa by proliferation of the epithelial cells. We found a 5-FU induced compensatory hyperproliferation in the recovery phase that took place 4 and 5 days after acute injury and that this hyperproliferation was associated with a marked increase in GLP-2 secretion.

Because both hormones have been shown to be intestinotrophic, we examined if single or co-treatment with both peptides in the same low dose (12.5 μg twice daily) could affect SI growth and found that in these lower doses GLP-1 did not elicit intestinotrophic effects alone but potentiated the effects of GLP-2, significantly increasing villus height and crypt depth in the duodenum and jejunum compared to GLP-2 alone. This finding supports a synergistic effect and a possibly better effect on mucositis with co-treatment. In groups treated with GLP-1, we noticed a significant weight loss after the first 24 h that normalised during the next 24 h. We decided to start treatment 2 days before the onset of mucositis in the next studies investigating the synergistic effects on mucositis. We found that in the selected dose, GLP-1 by itself had no effect and could not protect the mice from the weight loss due to mucositis or the loss of small intestinal mass. GLP-2 in single treatment showed marked protection against the total BW loss and a significant reduction in MPO in the ileum but very limited effect on intestinal weight and morphometric measures. When mice were treated with both peptides, we found protection from BW loss; a significant increase in small intestine weight, villus height and crypt depth; and a reduction in MPO. The effects of co-treatments were also significantly different from treatment with aGLP-2 alone–showing that co-treatment actually was superior to single treatment.

We used the Tg(GCG.DTR) mouse model, where the gene for human DT receptor is inserted after the proglucagon promoter, which makes proglucagon-expressing cells sensitive to injections with DT [22]. By measuring plasma GLP-2 at the end of the study, we could show that only the WT mice secreted GLP-2 as a consequence of mucositis. Our results showed very clearly that GLP-1 and GLP-2 deficient mice were not able to recover BW or SI weight after chemotherapy in the same manner as the WT mice. As demonstrated in study 1, the hyperproliferation during recovery shows up as an increase in crypt depth and this increase only occurred in the WT mice. We did not find any difference in the overall mucositis score, but looking solely at the crypts these were fully recovered in the distal intestine in the WT group but not in the Tg group. Together these experiments suggest that the compensatory hyperproliferation and intestinal healing depend on a functional L-cell secretion. When using the Tg(GCG.DTR) it must be taken into account that the glucagon-secreting pancreatic α-cells also experienced knockdown when DT is administered, and of course other L-cell products such as PYY are also diminished. To show that the L-cell products responsible for the proliferation and healing indeed are GLP-1 and GLP-2, we performed the experiments again, this time treating the Tg mice with either GLP-1 or GLP-2 or both. Co-treatment with both peptides restored BW and the co-treated Tg mice had a BW curve identical to that of WT mice. Importantly, a reduction in the histological scoring was seen in the WT mice as well as GLP-2 and co-treated mice. GLP-2 is a very potent growth hormone stimulating intestinal proliferation and the mechanism behind the protective effect of GLP-2 is also mirrored in the increased intestinal weight seen in the aGLP-2 and co-treated Tg mice. GLP-2 has been suggested to be responsible for the intestinal adaptive response [37] and this is in compliance with our results, showing that the compensatory hyperproliferation lacks in mice with an insufficient GLP-2 secretion and can be restored when GLP-2 is injected. GLP-2 has also been shown to protect the intestinal barrier in animal models of mucosal barrier dysfunction, such as jaundice [43], indomethacin induced enteritis [8] and obesity [44]. Despite the increase in small intestinal weight, the aGLP-2 treated mice suffered from a more than 15% BW loss, not different from Tg mice without substitution. In contrast to this, GLP-2 treatment in WT mice with mucositis affects BW in a positive manner (Study 2), but it must be emphasized that WT mice with mucositis still have the capacity to secrete GLP-1, as did mice in all the aforementioned studies examining mucosal barrier dysfunction. One could speculate that the GLP-2 effects on the intestinal barrier also need the presence of GLP-1.

Single treatment with GLP-1 is able to rescue a part of the severe weight loss in Tg mice, but with a modest effect on intestinal weight, indicating that several mechanisms are involved in intestinal healing and that GLP-1 and GLP-2 have different actions in the healing process. It is quite interesting that both hormones are needed in the healing process and from our results it could be hypothesized that GLP-1 could be important in a more systemic manner since it seems pivotal for restoring the BW. GLP-1 treatment has shown anti-inflammatory effects by suppressing pro-inflammatory cytokines in intestinal mucosa after induction of both dextran induced colitis in mice [45] and in a model of adoptive transfer colitis [46]. Our results do not directly support an anti- inflammatory effect, since myeloperoxidase, a marker of neutrophil granulocyte invasion could not be decreased with GLP-1 treatment alone; further experiments are needed to fully understand the mechanisms behind the protective effects of the hormones.

From these findings, we can conclude that at least in mice, a sufficient secretion of both GLP-1 and GLP-2 is essential for the healing process to occur.

From a clinical perspective, these results should be considered in supportive cancer care, where TPN should be used only in situations where nutrient supply is critical, because TNP will most likely prolong or abolish intestinal healing. Another clinical perspective to be considered is individual differences in L-cell function; for instance, patients suffering from type II diabetes have impaired secretion. Not all patients suffer from mucositis after chemotherapy and despite several epidemiological investigations the risk factors remain unknown. To our knowledge, diabetes or prediabetes has never been taken into account, and it could be speculated that an impaired L-cell function might be a risk factor of developing mucositis, rendering patients with diabetes or pre-diabetes more susceptible to chemotherapy. On the other hand obese subjects have impaired L-cell secretion as well, but obesity has been shown to be slightly protective against chemotherapy-induced mucositis [47;48]. This could, however, be due to a smaller effective dose because of the way doses are calculated as well as a change in distribution pattern in tissues [49–51] Whether the individual L-cell function in humans is critical for intestinal healing as a response to chemotherapy-induced mucositis needs further investigation.

## Supporting information

S1 Appendix DT substituted with BSA. To confirm that WT and Tg mice have the exact same phenotype and reactions to chemotherapy when not treated with diphtheria toxin (DT) we performed a study substituting DT with bovine serum albumin (BSA).
(PDF)

S1 Fig Study 1 The nature of mucositis induced with 5-FU. Mice were sacrificed 1–5 days after 5-FU injection at day 0. **a-b** area of BrdU immunopositive cells/crypt (μm2), **c-e** crypt depth (μm), **f-h** villus length (μm), **i-k** cross sectional area of mucosa (μm2). Results are shown as mean ± SEM n = 13. * = p < 0.05, ** = p <0.01 *** = p <0.001 compared to day 0 (ANOVA followed by Dunnett’s multiple comparison test).
(PDF)

S2 Fig Study 2 Single vs. co-treatment with Ex-4 and aGLP-2 in normal healthy mice. **a** Percent change in BW, **b-d** crypt depth (μm), **e-g** villus length (μm), **h-j** cross sectional area of mucosa (μm2). Results are shown as mean ± SEM n = 8. * = p < 0.05, ** = p < 0.01 compared to vehicle (Two-way ANOVA followed by a Bonferroni’s multiple comparison test (BW) or ANOVA followed by a Bonferroni’s multiple comparison test).
(PDF)

S3 Fig Study 3 Single vs. co-treatment with Ex-4 and aGLP-2 of acute mucositis. **a-c** crypt depth (μm), **d-f** villus height (μm), **g-I** cross sectional area of mucosa (μm^2^). Results are shown as mean ± SEM n = 6–8. * = p<0.05, ** = p<0.01 compared to healthy control (Vehicle), a = p<0.05 compared to 5-FU Vehicle (two-way ANOVA followed by a Bonferroni’s multiple comparison test (BW) or ANOVA followed by Dunnett’s multiple comparison test) b = p<0.05, bb = p<0.01 compared to Co-treatment (two-way ANOVA followed by a Bonferroni’s multiple comparison test (BW) or ANOVA followed by Bonferronis comparison test).
(PDF)

S4 Fig Study 4 The effect chemotherapy in GLP-1 and GLP-2 deficient mice. **a** Percent change in BW, **b** small intestinal weight (g), **c** plasma GLP-2 (μmol/l) **a-c** crypt depth (μm), **d-f** villus height (μm), **g-i** cross sectional area of mucosa (μm^2^). Results are shown as mean ± SEM n = 4–8. * = p < 0.05, ** = p < 0.01, compared to healthy control (WT Saline), a = p < 0.05, aa = p < 0.01 compared to WT 5-FU (ANOVA followed by Dunnett’s multiple comparison test).
(PDF)

S5 Fig Study 5 Single vs. co-treatment with Ex-4 and aGLP-2 inGLP-1 and GLP-2 deficient mice with mucositis. **a-c** crypt depth (μm), **d-f** villus length (μm), **h-j** cross sectional area of mucosa (μm^2^), **k-m** histological scoring of the small intestine. Results are shown as mean ± SEM n = 4–8. * = p < 0.05, ** = p < 0.01, compared to healthy control (WT Saline), a = p < 0.05, aa = p < 0.01 compared to WT 5-FU (ANOVA followed by Dunnett’s multiple comparison test).
(PDF)
