# Supplementary material for: Endogenous glucagon-like peptide- 1 and 2 are essential for regeneration after acute intestinal injury in mice
Source: PLoS One. 2018 Jun 4;13(6):e0198046. doi: 10.1371/journal.pone.0198046 (PMC5986149; doi:10.1371/journal.pone.0198046)
Supplement: S1 Appendix — To confirm that WT and Tg mice have the exact same phenotype and reactions to chemotherapy when not treated with diphtheria toxin (DT) we performed a study substituting DT with bovine serum albumin (BSA). (PDF) [file pone.0198046.s001.pdf]

S1 Appendix.

DT substituted with BSA

To confirm that WT and Tg mice have the exact same phenotype and reactions to chemotherapy when not treated with diphtheria toxin (DT) we performed a study substituting DT with bovine serum albumin (BSA).

Method

24 female Tgn(GCG.DTR) mice (20-25 g)[1] were used to rule out the effect of DT on body weight (BW) loss and SI weight. Instead of DT the mice were injected with BSA on day 0 and day 2. 5-fluorouracil (5-FU) was administrated on day zero. The mice were divided into four groups: Group 1: Tg 5-FU; group 2: Tg Saline; group 3: WT 5-FU; group 4: WT saline. BW was monitored every day and the mice were sacrificed 5 days after 5-FU administration. On the day of sacrifice the mice were anaesthetised with ketamine/xylazine 100/10 mg/kg i.p. After anaesthesia the mice were weighed, the abdomen was opened, the small intestine was removed and flushed with saline, excess saline was carefully removed, and the outer surface dried with paper before weight was recorded.

Results

There was no difference between Tg and WT mice with regard to BW loss and SI weight in both the saline-treated mice and the mice receiving chemotherapy. Mice given chemotherapy had a compensatory increase in small intestinal weight (% of BW) as seen in study 1.

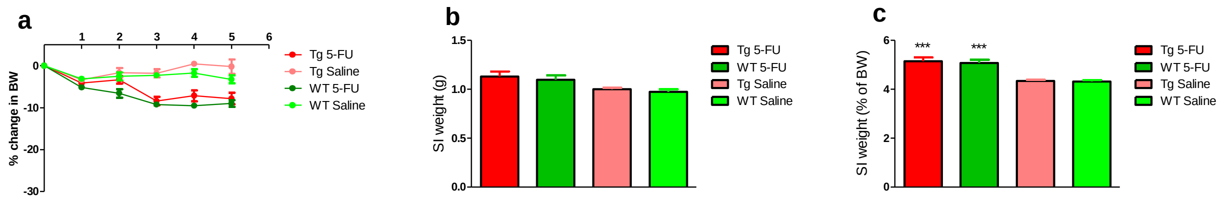

1. Pedersen, J., et al., *Glucose metabolism is altered after loss of L cells and alpha-cells but not influenced by loss of K cells.* Am J Physiol Endocrinol Metab, 2013. **304**(1): p. E60-73.
