# Supplementary material for: Endogenous glucagon-like peptide- 1 and 2 are essential for regeneration after acute intestinal injury in mice
Source: PLoS One. 2018 Jun 4;13(6):e0198046. doi: 10.1371/journal.pone.0198046 (PMC5986149; doi:10.1371/journal.pone.0198046)
Supplement: S1 Fig — Mice were sacrificed 1–5 days after 5-FU injection at day 0. a-b area of BrdU immunopositive cells/crypt (μm2), c-e crypt depth (μm), f-h villus length (μm), i-k cross sectional area of mucosa (μm2). Results are shown as mean ± SEM n = 13. * = p < 0.05, ** = p <0.01 *** = p <0.001 compared to day 0 (ANOVA followed by Dunnett’s multiple comparison test). (PDF) [file pone.0198046.s002.pdf]

S1 Figure Study 1 The nature of mucositis induced with 5-FU.

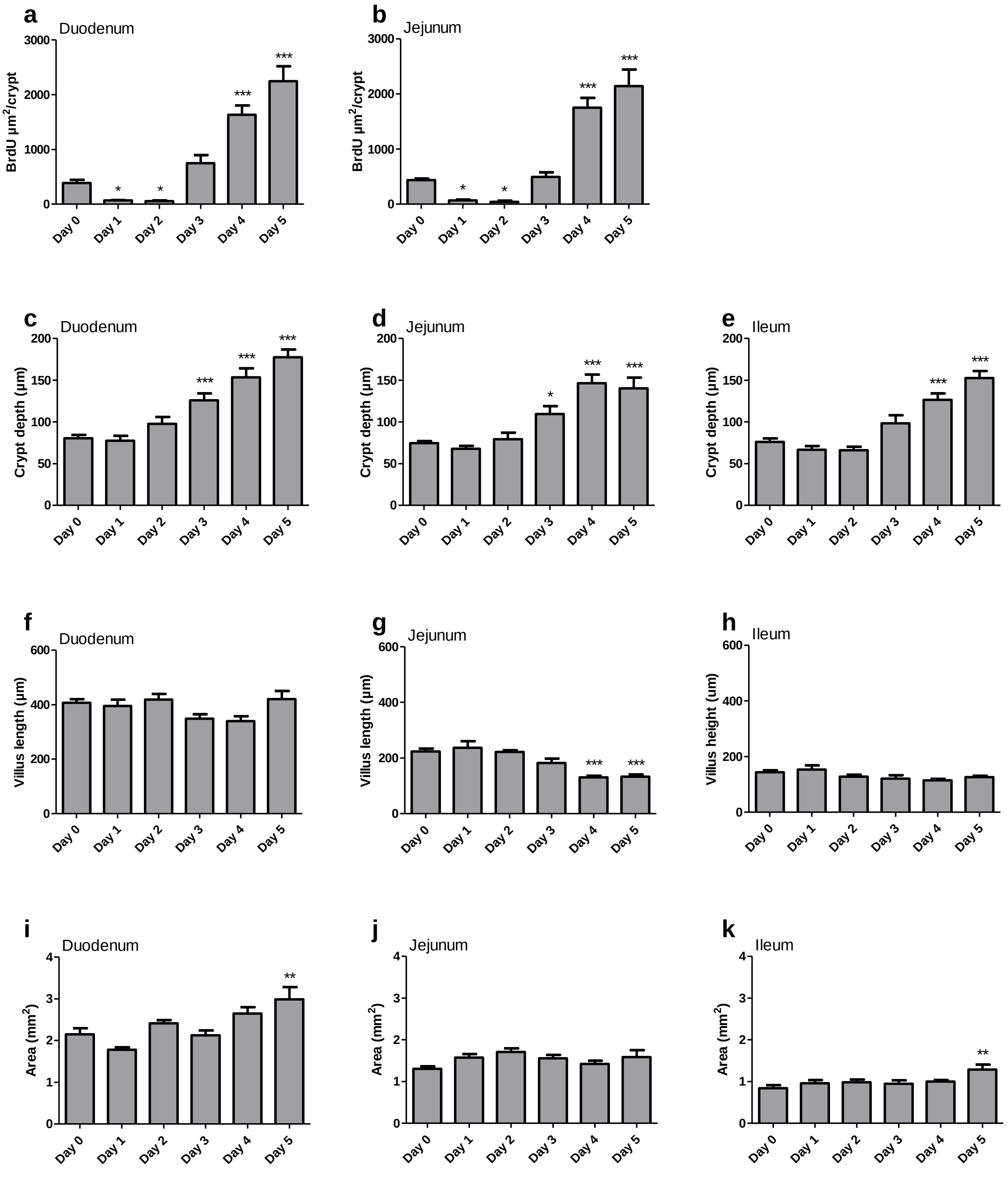

Study 1 The nature of mucositis induced with 5-FU. Mice were sacrificed 1-5 days after 5-FU injection at day 0. **a-b** area of BrdU immunopositive cells/crypt (μm2), **c-e** crypt depth (μm), **f-h** villus length (μm), **i-k** cross sectional area of mucosa (μm^2). Results are shown as mean ± SEM n = 13. * = $p < 0.05$, ** = $p < 0.01$ *** = $p < 0.001$ compared to day 0 (ANOVA followed by Dunnett's multiple comparison test).
