# Supplementary material for: Endogenous glucagon-like peptide- 1 and 2 are essential for regeneration after acute intestinal injury in mice
Source: PLoS One. 2018 Jun 4;13(6):e0198046. doi: 10.1371/journal.pone.0198046 (PMC5986149; doi:10.1371/journal.pone.0198046)
Supplement: S2 Fig — a Percent change in BW, b-d crypt depth (μm), e-g villus length (μm), h-j cross sectional area of mucosa (μm2). Results are shown as mean ± SEM n = 8. * = p < 0.05, ** = p < 0.01 compared to vehicle (Two-way ANOVA followed by a Bonferroni’s multiple comparison test (BW) or ANOVA followed by a Bonferroni’s multiple comparison test). (PDF) [file pone.0198046.s003.pdf]

S2 Figure. Study 2 Single vs. co-treatment with Ex-4 and aGLP-2 in normal healthy mice

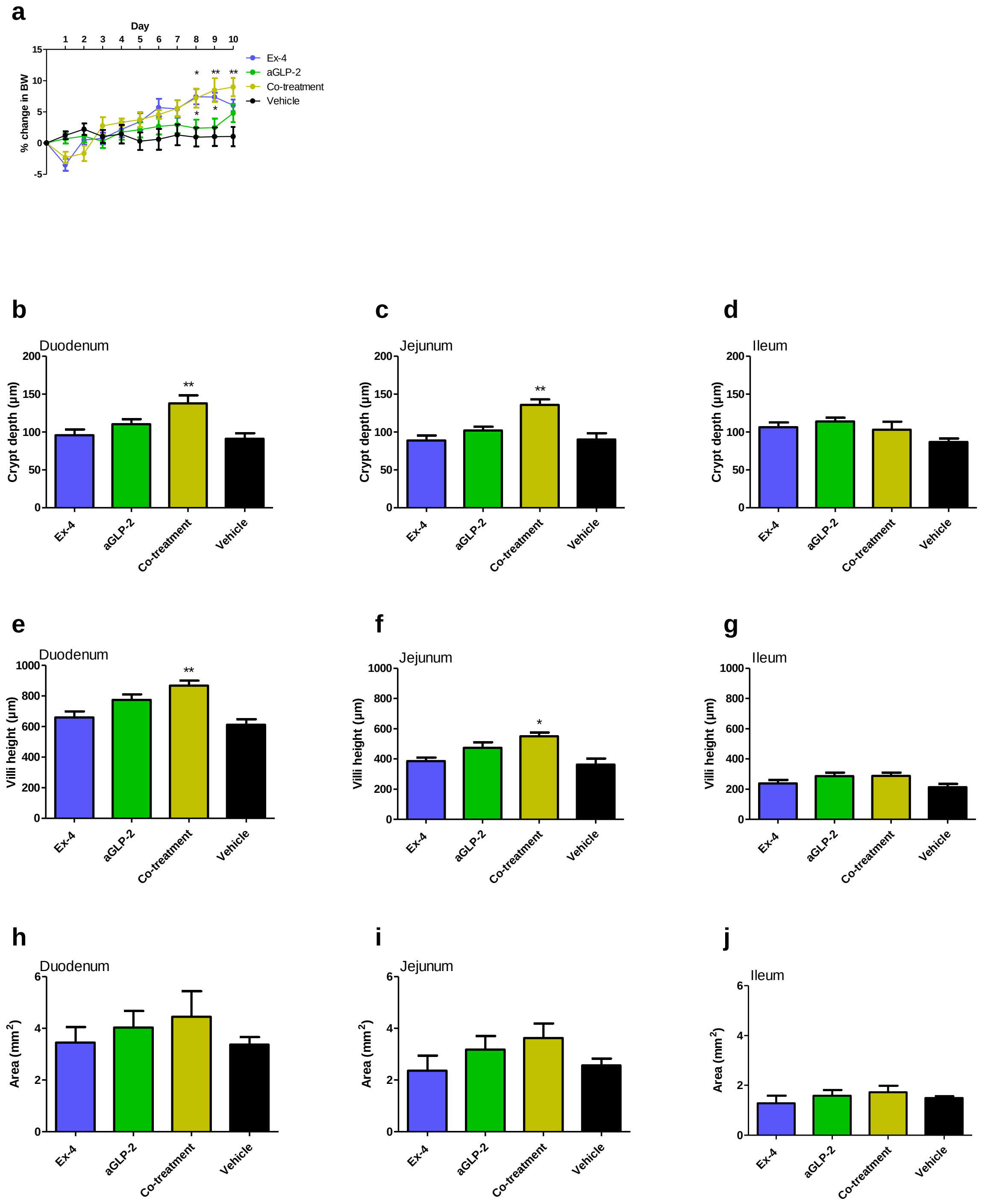

<u>**Study 2** Single vs. co-treatment with Ex-4 and aGLP-2 in normal healthy mice.</u> **a** Percent change in BW, **b-d** crypt depth (µm), **e-g** villus length (µm), **h-j** cross sectional area of mucosa (µm^2). Results are shown as mean ± SEM n = 8. * = p < 0.05, ** = p < 0.01 compared to vehicle (Two-way ANOVA followed by a Bonferroni's multiple comparison test (BW) or ANOVA followed by a Bonferroni's multiple comparison test).
