# Supplementary material for: Endogenous glucagon-like peptide- 1 and 2 are essential for regeneration after acute intestinal injury in mice
Source: PLoS One. 2018 Jun 4;13(6):e0198046. doi: 10.1371/journal.pone.0198046 (PMC5986149; doi:10.1371/journal.pone.0198046)
Supplement: S3 Fig — a-c crypt depth (μm), d-f villus height (μm), g-I cross sectional area of mucosa (μm2). Results are shown as mean ± SEM n = 6–8. * = p<0.05, ** = p<0.01 compared to healthy control (Vehicle), a = p<0.05 compared to 5-FU Vehicle (two-way ANOVA followed by a Bonferroni’s multiple comparison test (BW) or ANOVA followed by Dunnett’s multiple comparison test) b = p<0.05, bb = p<0.01 compared to Co-treatment (two-way ANOVA followed by a Bonferroni’s multiple comparison test (BW) or ANOVA followed by Bonferronis comparison test). (PDF) [file pone.0198046.s004.pdf]

S3 Figure

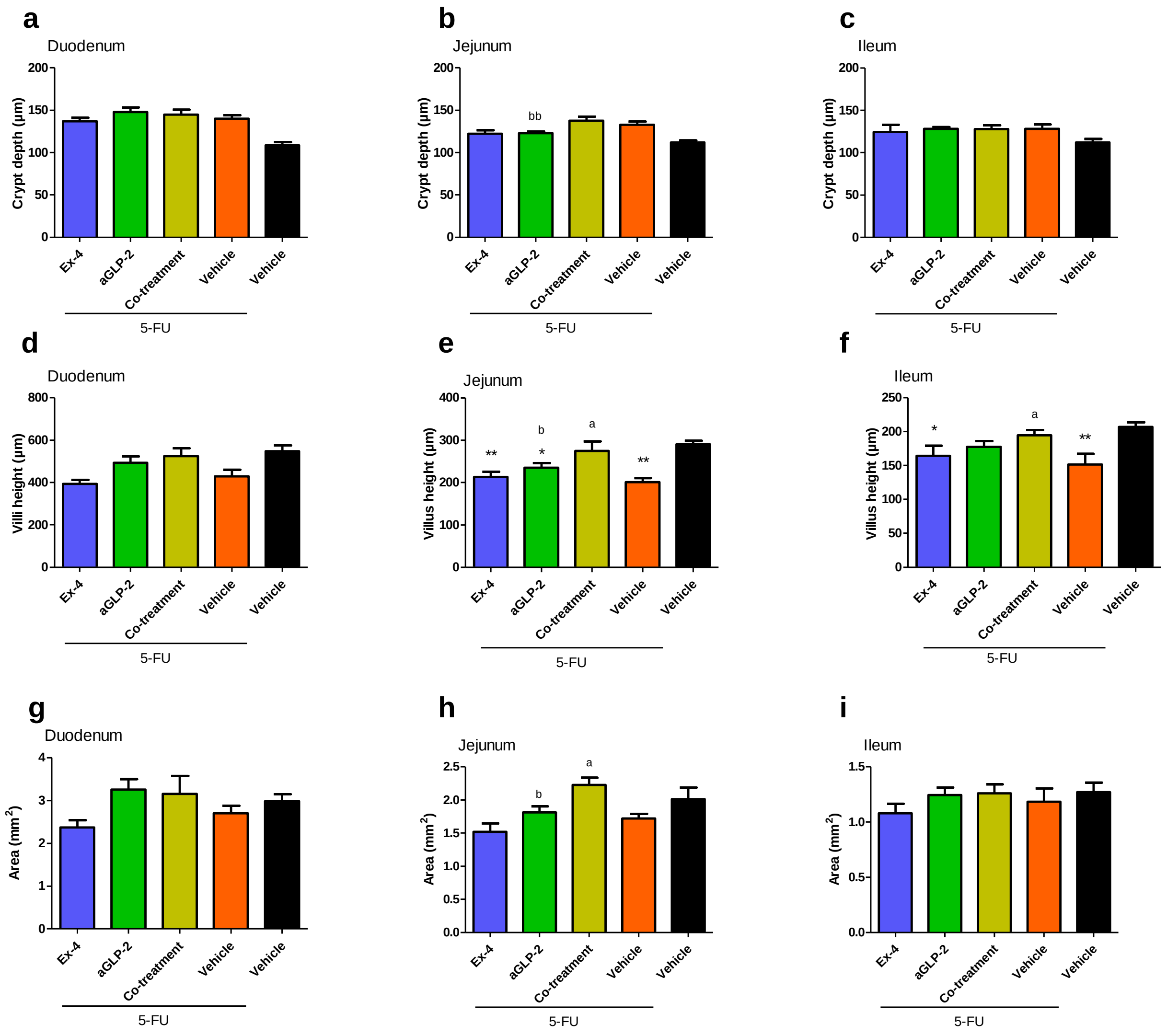

<u>**Study 3** Single vs. co-treatment with Ex-4 and aGLP-2 of acute mucositis</u>. **a-c** crypt depth (µm), **d-f** villus height (µm), **g-I** cross sectional area of mucosa (µm^2). Results are shown as mean ± SEM n = 6-8. * = p<0.05, ** = p<0.01 compared to healthy control (Vehicle), a = p<0.05 compared to 5-FU Vehicle (two-way ANOVA followed by a Bonferroni's multiple comparison test (BW) or ANOVA followed by Dunnett's multiple comparison test) b = p<0.05, bb = p<0.01 compared to Co-treatment (two-way ANOVA followed by a Bonferroni's multiple comparison test (BW) or ANOVA followed by Bonferronis comparison test)
