# Supplementary material for: Endogenous glucagon-like peptide- 1 and 2 are essential for regeneration after acute intestinal injury in mice
Source: PLoS One. 2018 Jun 4;13(6):e0198046. doi: 10.1371/journal.pone.0198046 (PMC5986149; doi:10.1371/journal.pone.0198046)
Supplement: S4 Fig — a Percent change in BW, b small intestinal weight (g), c plasma GLP-2 (μmol/l) a-c crypt depth (μm), d-f villus height (μm), g-i cross sectional area of mucosa (μm2). Results are shown as mean ± SEM n = 4–8. * = p < 0.05, ** = p < 0.01, compared to healthy control (WT Saline), a = p < 0.05, aa = p < 0.01 compared to WT 5-FU (ANOVA followed by Dunnett’s multiple comparison test). (PDF) [file pone.0198046.s005.pdf]

S4 Figure

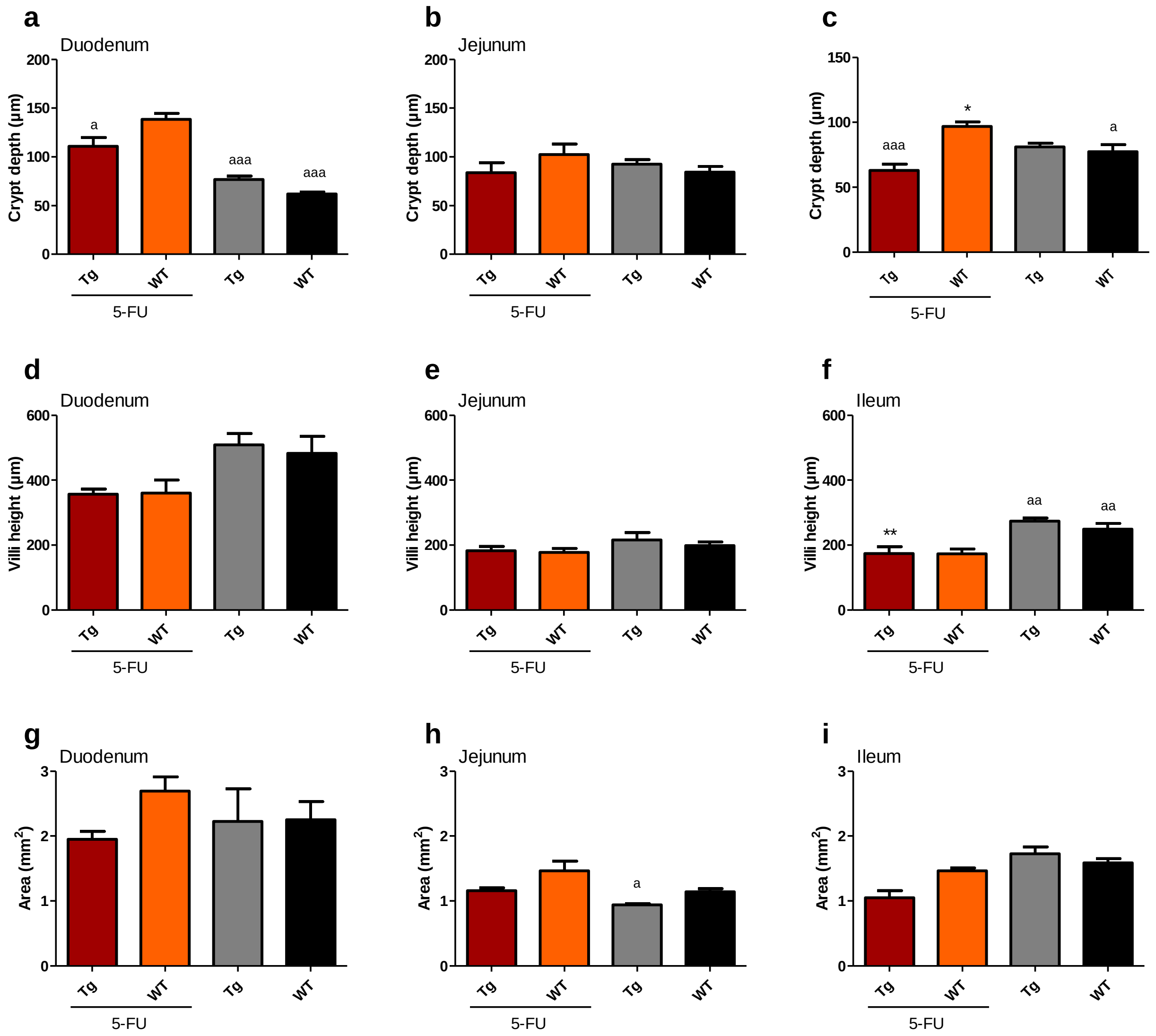

<u>**Study 4** The effect chemotherapy in GLP-1 and GLP-2 deficient mice.</u> **a** Percent change in BW, **b** small intestinal weight (g), **c** plasma GLP-2 (µmol/l) **a-c** crypt depth (µm), **d-f** villus height (µm), **g-i** cross sectional area of mucosa (µm^2). Results are shown as mean ± SEM n = 4-8. * = p < 0.05, ** = p < 0.01, compared to healthy control (WT Saline), a = p < 0.05, aa = p < 0.01 compared to WT 5-FU (ANOVA followed by Dunnett's multiple comparison test).
