# Supplementary material for: Endogenous glucagon-like peptide- 1 and 2 are essential for regeneration after acute intestinal injury in mice
Source: PLoS One. 2018 Jun 4;13(6):e0198046. doi: 10.1371/journal.pone.0198046 (PMC5986149; doi:10.1371/journal.pone.0198046)
Supplement: S5 Fig — a-c crypt depth (μm), d-f villus length (μm), h-j cross sectional area of mucosa (μm2), k-m histological scoring of the small intestine. Results are shown as mean ± SEM n = 4–8. * = p < 0.05, ** = p < 0.01, compared to healthy control (WT Saline), a = p < 0.05, aa = p < 0.01 compared to WT 5-FU (ANOVA followed by Dunnett’s multiple comparison test). (PDF) [file pone.0198046.s006.pdf]

S5 Figure

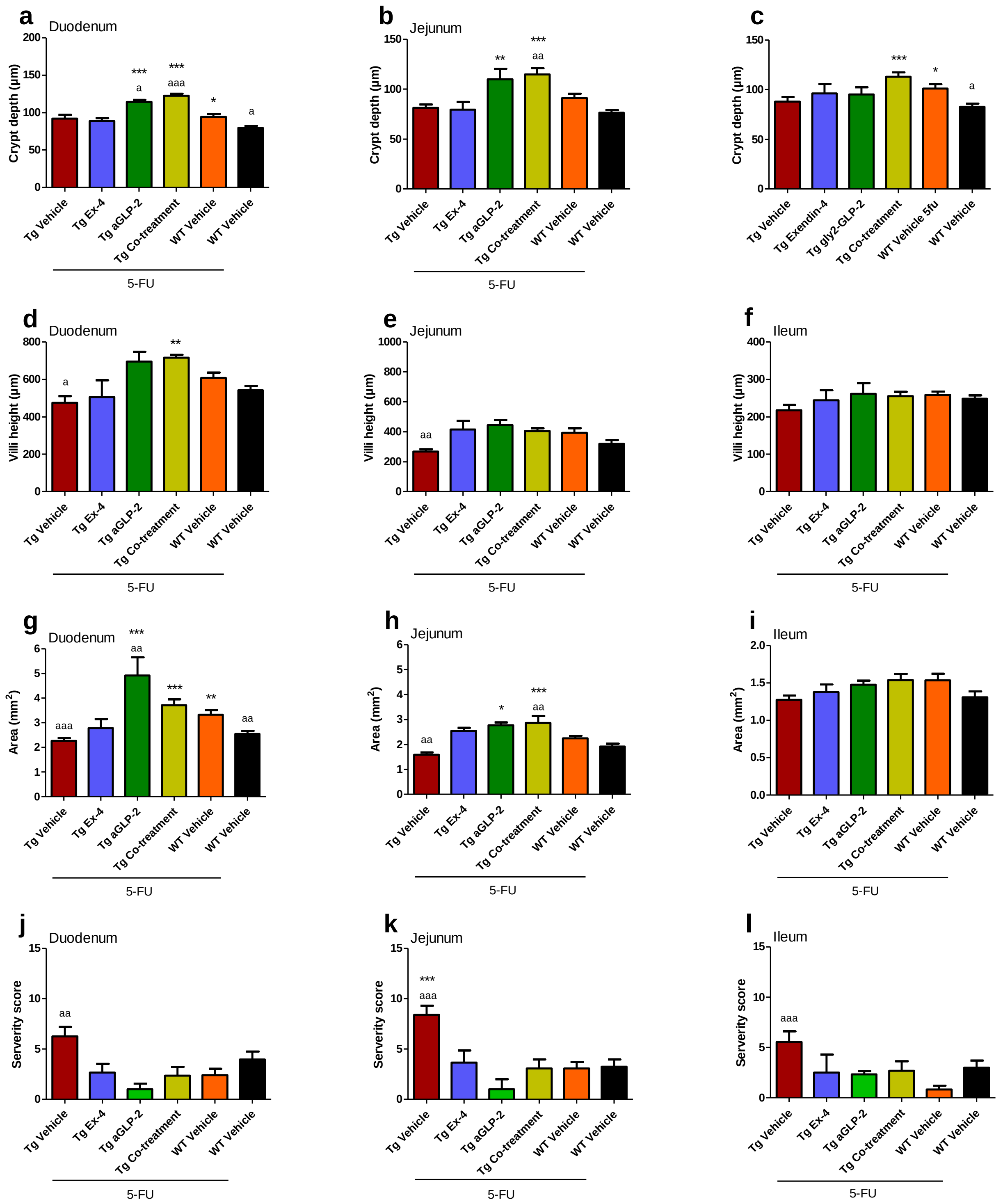

<u>**Study 5** Single vs. co-treatment with Ex-4 and aGLP-2 inGLP-1 and GLP-2 deficient mice with mucositis</u> **a-c** crypt depth (µm), **d-f** villus length (µm), **h-j** cross sectional area of mucosa (µm^2), **k-m** histological scoring of the small intestine. Results are shown as mean ± SEM n = 4-8. * = p < 0.05, ** = p < 0.01, compared to healthy control (WT Saline), a = p < 0.05, aa = p < 0.01 compared to WT 5-FU (ANOVA followed by Dunnett's multiple comparison test).
